# Pseudogenomic insights into the evolution of *Mycobacterium ulcerans*

**DOI:** 10.1186/s12864-024-10001-1

**Published:** 2024-01-22

**Authors:** Edwin Sakyi Kyei-Baffour, Kwabena Owusu-Boateng, Abiola Isawumi, Lydia Mosi

**Affiliations:** 1 West African Centre for Cell Biology of Infectious Pathogens, Department of Biochemistry, Cell and Molecular Biology, University of Ghana, Accra, Ghana; 2 Department of Microbial Sciences, University of Surrey, Surrey, UK

**Keywords:** Buruli ulcer, Evolution, Pseudogenes, Bioinformatic pipeline, Niche adaptation model

## Abstract

**Background:**

Buruli ulcer (BU) disease, caused by *Mycobacterium ulcerans* (MU), and characterized by necrotic ulcers is still a health problem in Africa and Australia. The genome of the bacterium has several pseudogenes due to recent evolutionary events and environmental pressures. Pseudogenes are genetic elements regarded as nonessential in bacteria, however, they are less studied due to limited available tools to provide understanding of their evolution and roles in MU pathogenicity.

**Results:**

This study developed a bioinformatic pipeline to profile the pseudogenomes of sequenced MU clinical isolates from different countries. One hundred and seventy-two MU genomes analyzed revealed that pseudogenomes of African strains corresponded to the two African lineages 1 and 2. Pseudogenomes were lineage and location specific and African lineage 1 was further divided into A and B. Lineage 2 had less relaxation in positive selection than lineage 1 which may signify different evolutionary points. Based on the Gil-Latorre model, African MU strains may be in the latter stages of evolutionary adaption and are adapting to an environment rich in metabolic resources with a lower temperature and decreased UV radiation. The environment fosters oxidative metabolism and MU may be less reliant on some secondary metabolites. In-house pseudogenomes from Ghana and Cote d’Ivoire were different from other African strains, however, they were identified as African strains.

**Conclusion:**

Our bioinformatic pipeline provides pseudogenomic insights to complement other whole genome analyses, providing a better view of the evolution of the genome of MU and suggest an adaptation model which is important in understanding transmission. MU pseudogene profiles vary based on lineage and country, and an apparent reduction in insertion sequences used for the detection of MU which may adversely affect the sensitivity of diagnosis.

**Supplementary Information:**

The online version contains supplementary material available at 10.1186/s12864-024-10001-1.

## Significance

Prevention and treatment of Buruli ulcer is still a problem but large whole genome datasets on *M. ulcerans* are readily available. However, genomic studies fail to thoroughly investigate pseudogenes to probe evolutionary changes in the bacteria, and this can be attributed to the lack of bioinformatic tools. This work studied pseudogenes in *Mycobacterium ulcerans* (MU) to understand its adapted niche and evolutionary differences across African strains. Our results posit an MU niche-adapted model important in understanding transmission. Also, MU pseudogene profiles vary based on lineage and country, suggesting their influence on pseudogenization patterns in the genome. We further identify a reduction in insertion sequences that are used for the detection of the bacteria which may affect the sensitivity of diagnosis.

## Background

The Mycobacterium genus consists of free-living pathogenic species like *Mycobacterium marinum*, host-adapted *M. tuberculosis, M. leprae*, and intermediates, *M. ulcerans* (MU) [1]. MU causes Buruli ulcer (BU), a neglected tropical skin disease characterized by necrotic and undermined lesions. Although the associated mortality rate is low, disease morbidity seriously affects the quality of life of affected persons because the massive scarring which occurs upon healing results in the contraction of affected parts [2]. BU cases saw an apparent decline over the past decade, however, there is a recent increase in cases globally [3]. Genomic studies have shown that MU may have evolved from an *M. marinum* common ancestor through the acquisition of a 174 kb plasmid encoding mycolactone; the established major virulence factor [4, 5]. The acquisition of the plasmid has led to the adaption to a new niche with the accumulation of pseudogenes and the reduction in the genome coding capacity [5, 6].

Pseudogenes are described as defective genes that have accumulated mutations (indels, frameshifts, and nonsense) or affected by the insertion of transposable elements [7]. Pseudogenes are less studied [8, 9], however, they can define the evolutionary trajectory, preferred environment, and survival strategies of bacteria [7, 10]. The lack of tools has hindered the profiling of pseudogenes [10], however, the availability of annotation tools like the DDBJ Fast Annotation and Submission Tool [11] has replaced manual curation methods. In addition, algorithms and computational approaches for identifying pseudogenes can facilitate characterization and functional studies of pseudogenes. Recently, the Prokaryotic Genome Annotator Pipeline [12] identified 1410 pseudogenes in the MU Agy99 strain (ASM1392v2) as compared to 771 previously reported [5]. Moreover, most studies on MU genomes have not focused on pseudogene accumulation and variations in the MU population [13–15].

We have developed a pipeline to predict and analyze the pseudogenomes of bacterial genomes to foster pseudogene analysis. We utilized the genome sequences of 172 MU clinical isolates, from Africa, with representative sequences from Australia, French Guiana, and the USA. Through pan-pseudogenome analyses, we show quantitative and qualitative variations across different MU isolates. We further propose a pseudogene-based niche model of MU and its implication on the transmission of BU.

## Results

### Pseudogenome dynamics among MU strains

#### Genome similarity/average nucleotide identity (ANI)

To understand pseudogene variation across the mycobacterial strains under study, the whole genome nucleotide similarities (Average Nucleotide Identity) of MU and *M. marinum* M (MmarM) strains were first determined using dRep. The ANI scores reported are based on the comparison between the *M. ulcerans* strains and their closest species *M. marinum* and we used the *M. marinum* M reference strain. With an Average Nucleotide Identity (ANI) threshold of 98.5% for the primary clusters, two [2] phylogroups were identified (Supplementary Figure S1). The large cluster consists of MU isolates (orange) with MmarM (blue) used as an outgroup. Comparing other non-African strains to the African strains, ANI for P7741 (French Guiana) was > 98.8%, 99.7% for the Australian isolate (MU496180), and 99.6% for Harvey (USA). For the in-house isolates, 99.9% was calculated for Z039 (Ivorian in-house strain) and > 99.8% for Z007. PA38 (Ghanaian in-house clinical strain) had the lowest similarity (> 99.4%) among the three in-house isolates when compared to the rest of the African strains. The MU strains were further divided in to two prominent clades; the purple-bounded box which had strains with a higher genome similarity to the reference strain Agy99, and the green-bounded box that shares an ancestor with the Agy99 clade (Supplementary Figure S1).

#### Pseudogene distribution across African strains

**Fig. 1:**
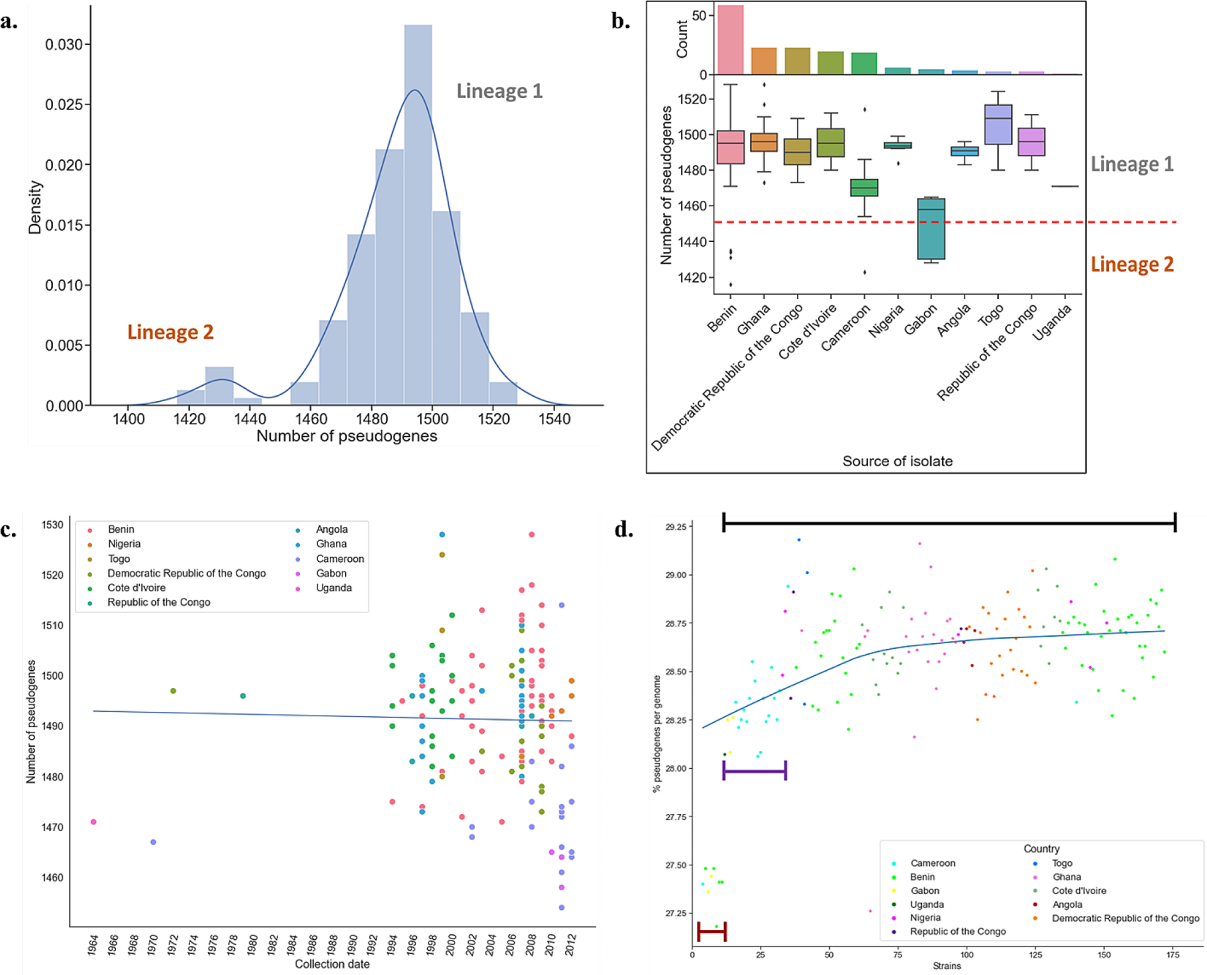
Distribution of pseudogenes across African MU strains. (**a**) Number of pseudogenes identified in the whole genome. (**b**) Distribution of the number of pseudogenes per country of isolate origin. The red dotted line indicates a boundary between the lineages based on the number of pseudogenes. Density is the frequency at which pseudogene numbers occurred. (**c**) Correlation between sample collection date and the number of pseudogenes in lineage 1 African strains. The blue line is a regression line with a negative Pearson’s rank correlation, r (156) = 0.022 (*p*-value = 0.78), and a gradient of -0.04 pseudogenes per year. Points are coloured by the source (country) of the strains. (**d**) Locally weighted scatterplot smoothing regression of the percent pseudogenes per MU genome. Strains were ordered starting with the closest isolate to the *M. marinum* reference to the farthest isolate according to their order in a maximum likelihood phylogenetic tree, and this is surrogate to for the root-to-tip distance. Black, purple, and brown- bounded lines represent lineage 1, the smaller sublineage of lineage 1 and lineage 2 respectively

The number of pseudogenes identified in the African population were within the range of 1420 and 1520. Two populations were identified, corresponding to the two MU lineages (1 and 2) with fewer pseudogenes in lineage 2 than in lineage 1 (Fig. 1a). Lineage 1 strains had a range of pseudogenes between 1429 and 1440 and lineage 2 between 1460 and 1520. Lineage 2 isolates were from Benin, Cameroon, and Gabon (Fig. 1b). Some isolates from Cameroon, Gabon, and Uganda had fewer pseudogenes as compared to the rest of the lineage one isolates (Fig. 1b).

#### Pseudogene accumulation in African strains over time

The number of pseudogenes in African MU strains was further analyzed for their correlation with the sampling date. In lineage 1 (r (154) = 0.022, *p*-value = 0.777) and lineage 2 (r [8] = 0.172, *p*-value = 0.683), there was no significant correlation (Fig. 1c). A phylogeny-based approach using locally-weighted regression across the two lineages showed that the percentage of pseudogenes per genome increased from lineage 2 to lineage 1 and then remains stable (Fig. 1d). These indicate that pseudogene accumulation may have stabilized, and the number of pseudogenes per MU genome and the sampling time may not be correlated.

#### Qualitative analysis of MU pseudogenomes

**Fig. 2:**
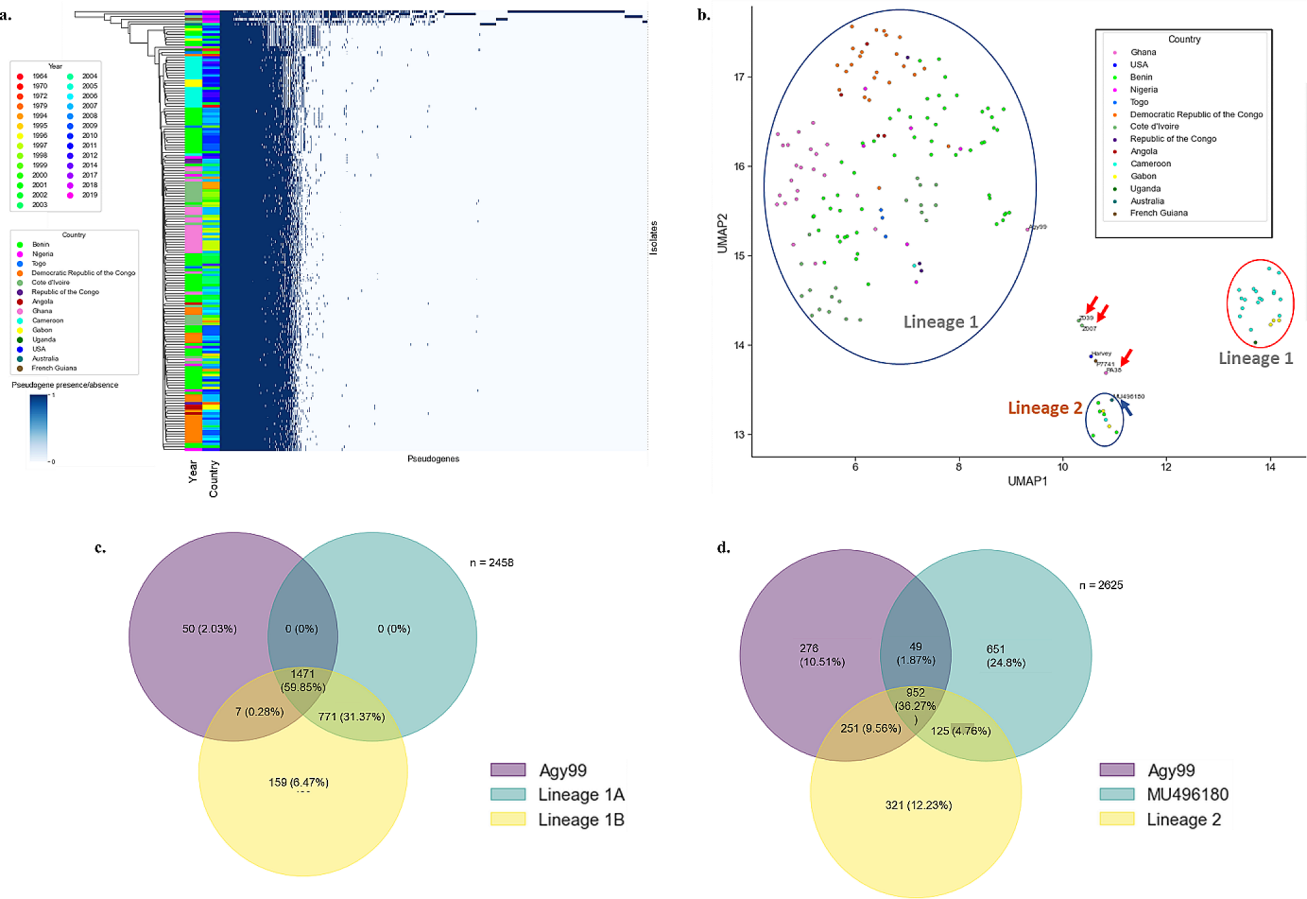
Clustering and phylogenetic analyses of pseudogenomes. (**a**) Comparison of pseudogenomes in *M. ulcerans* strains. Strains are coloured (left of the heatmap) based on the source (country), and on the immediate right, based on the year the sample was collected. The presence of a pseudogene is indicated by a blue bar and strains are clustered based on similarities in pseudogenome profiles (on the extreme left). (**b**) UMAP clustering of MU pseudogenomes. Isolates are coloured by country. Clusters are bounded in a blue line with lineage one and two clusters annotated. The red-bounded cluster represents a subcluster of lineage one with isolates from Gabon, Cameroon, and Uganda. Red arrows point to in-house isolates PA38, Z007, and Z039 while the blue arrow identifies the Australian strain. (**c, d**) Pie charts comparing the number of pseudogenes similar across Agy99, African lineage 1, African lineage 2 and the Australian strain MU496180

MU pseudogenomes were analyzed for the presence or absence of pseudogenes in their genomes. The patterns observed (Fig. 2a) were based on the origin (African and non-African) and lineages of the strains (Fig. 2a hierarchical cluster). Subclusters in lineage 1 were country-specific and this was observed for Cameroon (light blue)-Gabon (yellow) and Benin (light green)-Togo (deep blue) (Fig. 2a). Pseudogenization patterns were similar for isolates from specific African countries (Democratic Republic of Congo, Ghana), however, no patterns were observed for the sampling collection date (second heatmap in Fig. 2a).

**Fig. 3:**
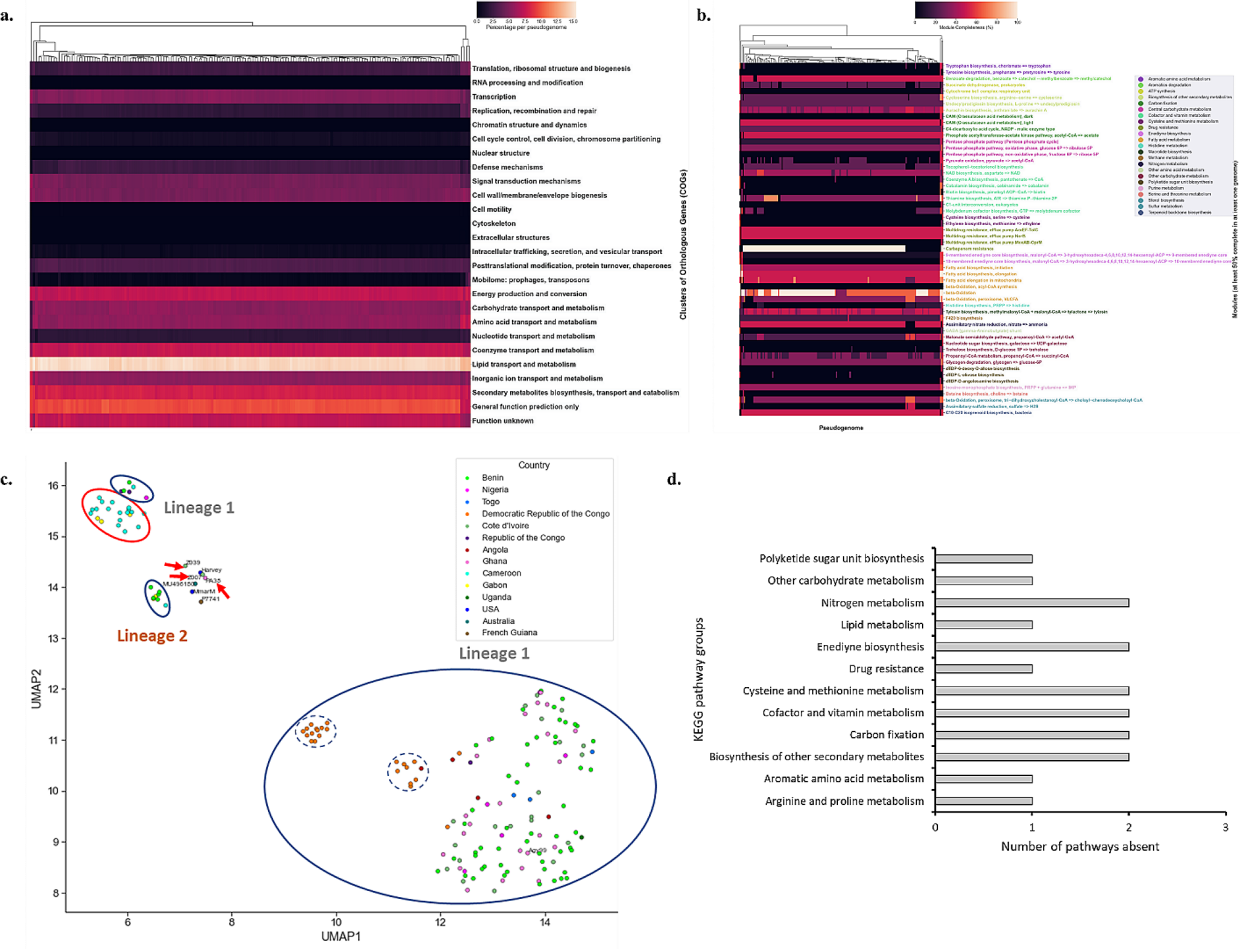
Cellular and metabolic analyses of pseudogenes in African MU strains. (**a**) Cellular processes of pseudogenes in MU strains using the COG database. The hierarchical cluster represents similarities in COG patterns across MU strains. Colours represent the percentage of pseudogenes per pseudogenome. (**b**) KEGG metabolic pathway analysis of pseudogenomes of MU strains [50]. Pathway modules are coloured based on the broader metabolism groups and each pathway module has an *M. ulcerans* strain that shows at least ≥ 50% module completeness. The hierarchical cluster represents similarities in metabolic patterns across MU strains. (**c**) UMAP clustering of module completeness of pseudogenomes of MU strains. Strains are coloured by known origin. Clusters are bounded by a blue line with strains from lineage 1 and 2 annotated. Broken blue circles are subclusters of the larger lineage 1 cluster. The red-bounded cluster represents a lineage 1 subcluster with isolates from mainly Gabon and Cameroon. Red arrows indicate in-house isolates PA38, Z007, and Z039. (**d**) Distribution of KEGG metabolic pathways completely pseudogenized across the African MU strains

Further dimensionality reduction analysis with UMAP confirmed the hierarchical clustering (Fig. 2b). However, lineage 1 was further broken down into two different clusters. The bigger cluster, lineage 1 A (bounded in blue, Fig. 2b), had majority of the isolates in lineage 1 while the smaller cluster, lineage 1B (bounded in red, Fig. 2b), had isolates from Cameroon (light blue)-Gabon (yellow), and Uganda (deep green). Isolates from Gabon and Cameroon had more pseudogenes in common than when individually compared to Uganda (Fig. 3 Cameroon (light blue)-Gabon (yellow) subclusters).

Lineage 2 (bounded in blue in Fig. 2b) was represented by isolates from Benin, Cameroon, and Gabon. To highlight the diversity in pseudogenes across lineages 1 A and 1B, they were compared to Agy99 reference strain. Lineage 1 A, 1B and Agy99 had 1471 pseudogenes in common (Fig. 2c). Lineage 1 A had no unique pseudogenes but had an additional 771 pseudogenes common to only lineage 1B. Lineage 1B had the most unique pseudogenes (159) sharing 7 additional pseudogenes with only Agy99. This indicates the diversity in lineages 1B compared to 1 A though both lineages have most pseudogenes in common. The Australian strain (MU496150) clustered with the African lineage 2 strains (Fig. 2b and Supplementary Figure S2) but lineage 2 share more pseudogenes (251) with Agy99 in addition to pseudogenes common to all three (Fig. 2d). However, a high number of pseudogenes was unique (321) to lineage 2 as well as pseudogenes (125) shared with only MU496150.

In-house sequenced clinical strains clustered away from the African lineages (Fig. 2b), which indicates differences in pseudogene profiles. The Ivorian isolates Z039 and Z007 shared pseudogenes with both African (251) and non-African (255) strains (especially Harvey and P7741) (Supplementary Figure S3). However, PA38, shared 165 pseudogenes with the African strains compared to 523 for the non-African strains and had the highest number (2885 and 2530) of unique pseudogenes among the three in-house isolates (Supplementary Figure S3). Pseudogenes similar to the African strains and the in-house strains were 1209 compared to 1370 pseudogenes in the non-African strains.

Pseudogenes peculiar to the African group include ESAT proteins (*EsxP, EsxT*), the trehalose transport system permease protein (*SugA*), and the Sulfate adenylyl transferase protein. Pseudogenes specific to the non-African strains include the siderophore export accessory protein (*MmpS4*), carbonic anhydrase 2, and the acid resistance serine protease (*Marx*). Pseudogenes in only Z007 include the chlorite dismutase and the *TlpA* family protein disulfide reductase. Nitrate/nitrite transporter (*NarU*) and the ESX-5 secretion system protein (*EccD5*) were identified in only Z039 while the DNA replication and repair protein (*RecF*) and the resuscitation-promoting factor (*RpfC*) were identified in only PA38.

#### Phylogenetic analysis on MU isolates

Further phylogenetic analysis showed that the Ivorian and Ghanaian in-house strains were part of lineage 1 sharing ancestors with other African strains from Côte d’Ivoire, Benin, and Ghana. PA38 had a larger genome (Prokka: CDS = 10,156; DFAST: CDS = 8505) than expected for African strains (Agy99 reference: CDSs = 5,193) and similar to the American strain Harvey (CDS = 9,177). PA38 also had more base substitutions (length of tree branch in Supplementary Figure S5) compared to its closest branch (Benin strain MU3223964) in the clade. The small lineage 1 subcluster formed a single clade, having a common ancestor with the large subcluster, however, the Ugandan strain had a common ancestor with the lineage 1 clade.

#### Selection pressure on MU pseudogenomes

**Fig. 4:**
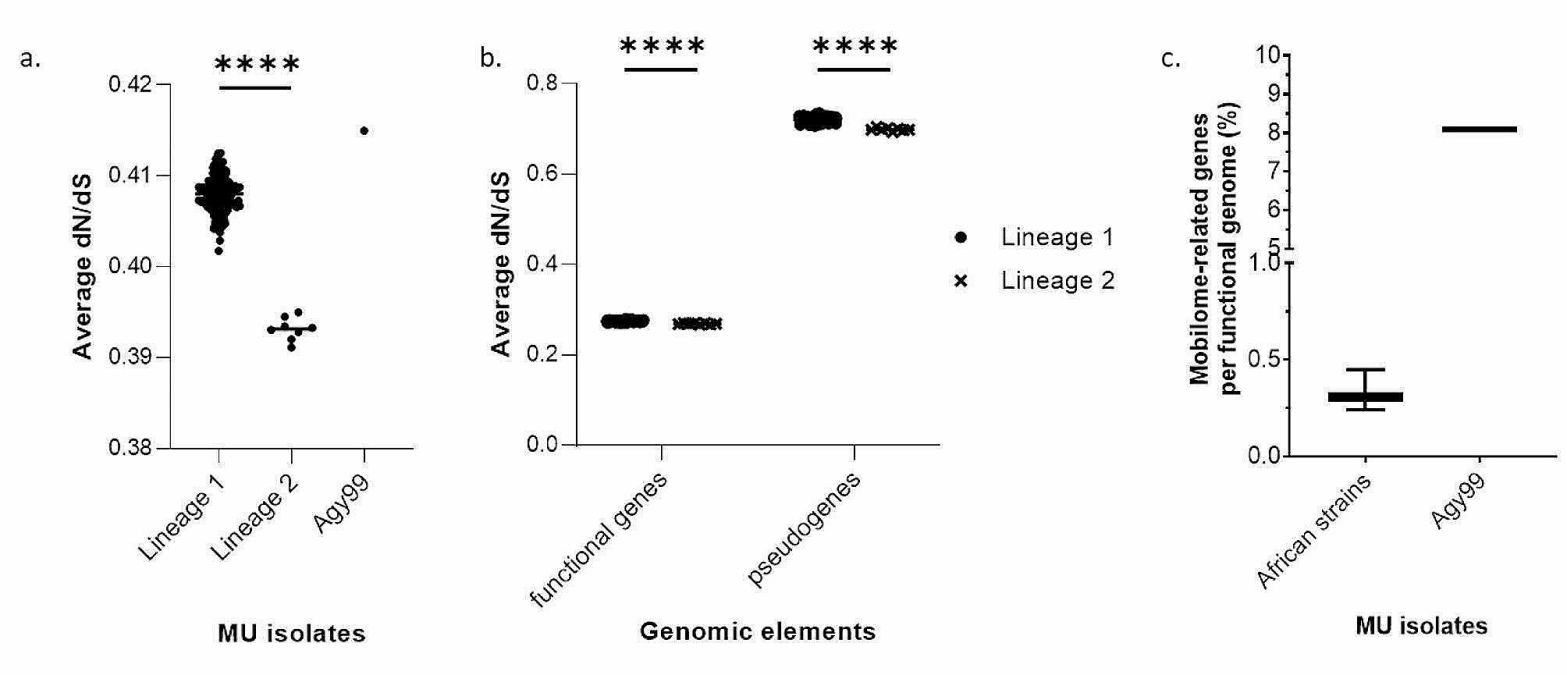
dN/dS mobilome analyses among the different M. ulcerans lineages and genomic elements present in M. ulcerans genomes. (**a**) Each point represents the average dN/dS value of an isolate and is grouped based on lineage. Mann-Whitney’s test was performed between lineage 1 and 2 and **** represents a *p*-value < 0.0001. (**b**) Each point represents the average dN/dS value of an isolate and is grouped based on lineage and the type of genomic element. Mann-Whitney’s test was performed between lineage 1 and 2 for each genomic element, and **** represents a *p*-value < 0.0001. (**c**) Distribution of mobilome-related genes and insertion sequence in African M ulcerans strains. The bar represents the distribution of the percentage of mobilome related-related genes in the functional genome. Only a single value is reported for the reference strain Agy99

Selection pressure was determined by the ratio of non-synonymous mutations (dN) to synonymous mutations (dS)). A dN/dS value < 1 represents negative selection, > 1 positive selection, and = 1 for neutral selection [16]. Selection pressure on the pseudogenome showed similar lineage division across the African isolates (Fig. 4a). Lineage 1 (pseudogenome = 0.719–0.72, functional genome = 0.27–0.28) had decreased dN/dS compared to lineage 2 (pseudogenome = 0.693–0.704, functional genome = 0.267–0.27), showing the decrease in non-synonymous mutations (dN) in lineage 2 compared to lineage 1 (Fig. 4b). Pseudogenomes (0.713 ± 0.032) showed a relaxed negative selection compared to the functional genes (0.275 ± 0.009) which represents the whole genome without pseudogenes (Fig. 4b).

PA38 had a very high dN/dS for the whole (0.49923) and functional (0.381384) genomes compared to the African MU strains (whole genome = 0.4079 ± 0.00213, functional genome = 0.2746 ± 0.00165). However, the pseudogenome was lower (0.532772) than the African strains (0.713 ± 0.032), indicating the role of the functional genome in increasing the whole-genome dN/dS. The whole genome dN/dS for the Ivorian in-house isolates was similar (Z007 = 0.417055, Z039 = 0.415869) to that of lineage 1, although slightly higher (Z007 = 0.29378, Z039 = 0.291345) for the functional genome and lower (Z007 = 0.514237, Z039 = 0.584934) for the pseudogenome. For the non-African isolates, Harvey had the highest (0.294238) and lowest (0.47915) functional genome and pseudogenome dN/dS respectively. Also, P7741 (French Guiana) had the highest (0.681232) and lowest (0.256212) dN/dS values for the pseudogenome and functional genome respectively. The Australian strain, MU496180, had dN/dS values (whole genome = 0.381127, functional genome = 0.264045, pseudogenome = 0.61921) which were between P7741 and Harvey.

#### Evolved African MU strains and PA38

The genome characteristics of the African MU strains and PA38, as summarized in Table 1 shows that the African MU strains in this study had a reduced genome (African MU strains = 5,240,186 ± 23,883 bp) with reduced gene-coding capacity (4005 ± 42 genes) compared to *M. marinum* M (6,660,144 bp, 5654 genes). This can be attributed to the accumulation of a high number of pseudogenes (1494 ± 80) and further genome erosion (MU strains = 5,240,186 ± 23,883 bp, *M. marinum* M = 6,660,144 bp). However, in PA38, there was a high number of pseudogenes (5617) (Supplementary Figure S3) with no observable genome erosion (PA38 = 5,837,271 bp, African MU strains = 5,240,186 ± 23,883 bp, *M. marinum* M = 6,660,144 bp). Also there was further loss of the mobilome (9 ± 16) (Fig. 4c) and increased purifying selection (Fig. 3). In PA38, there was an expanded genome (CDS = 10,156), a high mobilome (mobilome = 134), and decreased purifying selection, especially in the functional genome (dN/dS = 0.381384) (Fig. 3). This highlights the two contrasting characteristics of the evolved MU genomes with reference to *M. marinum* M.

**Table 1:** Pseudogenome and functional genome characteristics of African and PA38 MU strains

Genome characteristics	African isolates	PA38
Genome reduction	Yes	Yes
Expanded genome	No	Yes
Decrease in gene-coding capacity	Yes	Yes
Increased IS elements	No	Yes
Decreased purifying selection	No	Yes
Increased pseudogenes levels	Yes	Yes
Further genome erosion	Yes	No
Loss of mobilome (IS elements)	Yes	No
Increased purifying selection	Yes	No

#### Cellular and metabolic pathways of pseudogenes

In analyzing the cellular processes of pseudogenomes in the MU strains with the Clusters of Orthologous Genes (COG) database, most pseudogenes were involved in lipid transport and metabolism (14.66% ± 0.31%). Other cellular processes included general function prediction only (genes with poorly characterized function) (9.67% ± 0.41%), secondary metabolites biosynthesis (8.1% ± 0.24%), and pseudogenes with unknown function (7.28% ± 0.37%) (Fig. 3a). There was minimal variation in COG profiles across the African MU lineages (t = -0.005, *p*-value = 0.996). However, COG groups such as defense mechanisms showed more pseudogenes for lineage 1 strains (2.69% ± 0.11%) compared to lineage 2 (1.93% ± 0.09%) (Fig. 3a). The lack of variation observed in the functional genome between the lineages (t = -10.506, *p*-value = 7.348) (Supplementary Figure S4), indicates that the additional defense-related pseudogenes present in lineage 1 may have further been lost.

In-house clinical strains had similar COG patterns compared to the Harvey strain. These include the cell wall/membrane/envelope biogenesis class (In-house = 5.37%, Harvey = 5.8%), and signal transduction mechanisms class (In-house = 3.68%, Harvey = 3.62%). COGs that were similar between the African and the in-house strains include energy production and conversion (7.27% ± 0.72, 7.39% ± 0.16), and inorganic ion transport and metabolism (4.92% ± 0.23%, 5.47% ± 0.13%) (Fig. 3a). However, there was pseudogenization of mobilomes (including transposons) in the in-house strains (1.88% ± 1.79%) compared to the African strains (0.31% ± 0.1%) with Z039 having the least (0.68%).

The metabolic functions of pseudogenomes of the MU strains was determined with the KEGG database (Fig. 3b). Pathway variations were observed in twenty-five different metabolic pathways (Supplementary Table S1). Prominent metabolic pathways that showed differences between the strains (Fig. 3b) include the succinate dehydrogenase (prokaryotes), carbapenem resistance, molybdenum cofactor biosynthesis (GTP to molybdenum cofactor), histidine biosynthesis (PRPP to histidine) and assimilatory sulfate reduction (sulfate to H_2_S).

Twenty-five metabolic pathways were affected by pseudogenization (Supplementary Table S1). However, pathways that were more affected across the MU strains include the biosynthesis of secondary metabolites (undecylprodigiosin biosynthesis), carbon fixation (phosphate acetyltransferase-acetate kinase pathway), cofactor and vitamin metabolism (NAD biosynthesis), drug resistance (carbapenem resistance), fatty acid metabolism (beta-oxidation) and other carbohydrate metabolism (malonate semialdehyde pathway). Other metabolic processes affected include aromatic degradation (benzoate degradation), ATP synthesis (succinate dehydrogenase), macrolide biosynthesis (tylosin biosynthesis), nitrogen metabolism (assimilatory nitrate reduction), sterol (beta-oxidation, peroxisome) and terpenoid backbone biosynthesis (C10-C20 isoprenoid biosynthesis, bacteria). These highlight the metabolic changes that have occurred in the African strains.

Further clustering across the strains identified clusters corresponding to the African MU lineages (Fig. 3c). However, the larger African lineage 1 cluster had subclusters (boundary with a broken line, Fig. 3c) with strains from the Democratic Republic of the Congo and Angola. The smaller African lineage 1 cluster (bounded in red, Fig. 3c) was joined by strains (Nigeria, Benin, Cameroon, and the Republic of Congo) from the large lineage 1 cluster. Lineage 2 strains (labelled and blue-bounded in Fig. 3c) clustered closely to the non-African isolates. The in-house isolates (red arrows in Fig. 3c) did not cluster with any of the African lineages (red arrows in Fig. 3c). This highlights the correspondence between the metabolic and pseudogene absence-or-presence analyses.

To determine the impact of pseudogenization on the whole genome, the functional genome was compared to the whole genome for pathways completely or partially pseudogenized (Supplementary Figure S6, Supplementary Figure S7, Supplementary Figure S8). Two KEGG metabolism groups; nitrogen metabolism and polyketide sugar biosynthesis were absent in the functional genome (Supplementary Table S1). Eighteen pathways were completely pseudogenized across the African MU strains (Fig. 3d); two pathways were lost in nitrogen metabolism, enediyne biosynthesis, cysteine and methionine metabolism, cofactor and vitamin metabolism, carbon fixation, and biosynthesis of other secondary metabolites. Polyketide sugar unit biosynthesis, other carbohydrate metabolism, lipid metabolism, drug resistance, and aromatic amino acid metabolism lost one pathway each.

## Discussion

In this study, pseudogenomes of different clinical MU strains were studied to understand the dynamics of pseudogenes and the evolution of the bacteria, using a custom pipeline that was developed to identify and analyze the bacterial pan-pseudogenome. The pipeline does not only provide current and readily available pseudogene identification tools (Prokka, DFAST, and Pseudofinder) but also provides a combined and robust output of pseudogenes for each bacterial genome while reducing replicates. Further analyses (pseudogene distribution and clustering, COG clusters, and KEGG metabolic pathways) by the pipeline identifies pseudogenes to help delineate the impact of pseudogenization on the whole genome. This provides deeper insights into genome evolution in bacterial populations as compared to current tools that identify pseudogenes [17, 18]. Also, it removes the need for raw sequence reads preprocessing before analysis as present in Pseudofinder, and each stage in the pipeline can be run individually making it a flexible all-in-one tool for studying bacterial pseudogenes.

The pipeline was used to study mycobacterial genomes from different countries. Lineage- and country-specific clustering of mycobacterial strains based on their pseudogenomes indicate the influence of ancestry on the pseudogenome. For African MU strains, lineage 1 emerged from an ancestral strain, which spread and occupied certain African regions [13] that has allowed African MU strains to persist [19, 20]. The thriving of these strains in these areas may have led to differential pseudogenization signatures of these different countries. The introduction of a second lineage into Africa [13] further highlights the adaptation of the bacteria to Western and Central Africa as these strains had unique and shared pseudogenes with both lineage 1 and the non-African MU strains. However, the role of lineage 2 in disease burden and transmission in Africa is unknown. The clustering of the Australian isolate with lineage 2 may require further a larger investigation to delineate the relationship between these two geographically distinct MU strains.

Although the in-house isolates were MU strains from Africa lineage 1 and shared pseudogenes with the African isolates, their pseudogenome pattern was less similar to the African MU strains, especially PA38. Multiple loci VNTR genotyping shows that Z007, Z039, and PA38 have the common African profiles C and D [21]. PA38 further clustered with a strain from Benin pointing to the possible origin of the PA38 strain. The variations observed may be due to strain diversity that may have emerged in the sampled endemic communities [15, 22].

Based on the evolution of endosymbiont bacteria [7], the African MU strains are in stage two of evolutionary adaptation after pMUM plasmid acquisition. This was characterized by increased pseudogene numbers, further genome erosion compared to *M. marinum* M strain, the loss of insertion sequences, increased purifying selection (decreasing non-synonymous mutation), and the decrease in pseudogenes in both African lineages. The loss of insertion sequences (IS*2404*) in the second stage has implications for the diagnosis of BU because of its use in diagnosis [23]. This requires further studies into the variations in the number of IS*2404* and other insertion sequences in the genome, and how their elimination can impact diagnosis to inform better diagnostic strategies. The Ghanaian strain PA38, however, had an expanded genome, decreased genome-coding capacity, increased insertion sequence elements, and decreased purifying selection suggesting that it was in the first stage of evolutionary adaptation.

Metabolic variations observed across the MU strains identified multiple pathways including biosynthetic pathways (sterol and macrolide biosynthesis) partially or completely lost due to pseudogenization. This indicates the reduction in biosynthetic pathways that may not be currently essential for the bacteria [7]. The pseudogenization of fatty acid metabolism-related genes indicates the elimination of a nonessential subset of these genes, however, MU has a large number of genes related to fatty acid metabolism [24]. The loss of the shikimate pathway, disruption of propionyl coA, and nitrate metabolism [5] were confirmed in this study.

Based on the pseudogenization of metabolic pathways across the MU strains, a niche model was generated. This proposed niche shields the bacteria from UV radiation due to the loss of the undecylprodigiosin production pathway [25] in addition to the loss of the *crtB* gene [5] This niche fosters oxidative metabolism due to the loss of the dissimilatory nitrate reduction metabolic pathway that serves as an electron acceptor under anaerobic conditions [26]. The niche has a lower temperature as compared to its previous environment due to the loss of the Crassulacean acid metabolism (CAM) cycle light stage which is important for adapting to warm temperatures [27]. This is observed in the growth temperature (29–33℃) of the bacteria [2] and the disease mostly affecting the extremities of the body [28, 29]. Also, there is the availability of metabolic resources like amino acids (arginine) and cofactors (coenzyme A) due to the loss of these pathways [10]. This niche either has less competition [30] and/or the acquisition of mycolactone has allowed for the reduction of other secondary metabolites [5]. Secondary metabolites lost include enediynes (cause cell death) [31], tylosin (a macrolide antibiotic) [32] and the loss of resistance-mediated pumps (*MexJK-OprM* multidrug efflux pump that mediates resistance to tetracyclines and macrolides) [33].

With the identification of seven metabolic pathways unaffected by pseudogenization across MU strains, four pathways were found to have viable drug targets through literature search. The four pathways were selected based on the importance of the pathway to the bacteria and its absence in humans [34]. These pathways include biotin synthesis which has the BioA enzyme important for *M. tuberculosis* infection and persistence in tuberculosis. Compounds that inhibit BioA in *M. tuberculosis* include amiclenomycin [35] and N-aryl, N′-benzoylpiperazine- based compounds [36]. Another pathway is the betaine biosynthesis pathway. Betaine serves as an osmoprotectant and some targets include BetA, BetB, and BetL [37, 38]. Other pathways and their possible targets include tryptophan biosynthesis enzymes (TrpE, TrpA, and TrpB) [39] and essential beta-oxidation enzymes [24]. For the tryptophan biosynthesis pathway, indole propionic acid inhibits TrpE while a synthetic azetidine inhibits TrpA [39].

## Conclusions

This study developed a pipeline to investigate the variations in MU pseudogenomes but can be extended to other bacteria to identify and analyze their pseudogenomes. It is was observed that MU pseudogenomes are lineage- and location-specific. African MU strains are in the second stage of evolutionary adaptation, however, there are variations which are lineage-dependent. A proposed pseudogene-based MU niche model highlights a niche that protects it from UV radiation and fosters oxidative metabolism with relatively lower temperature, availability of metabolic resources, and has less reliance on secondary metabolites. The in-house clinical strains differed in pseudogenomic signatures compared to the African population, however, phylogenetic relationships and VNTR genotyping highlight their close relationship with African MU strains. Also, the PA38 strain may be in the early stages of evolutionary adaptation. The highlighted pathways and targets unaffected by pseudogenization can be further studied for alternative therapeutic targets for BU treatment. In summary, this tool provides the ability to investigate pseudogenomes in bacterial populations to generate insights and hypotheses for further investigation.

## Methods

### Study design and strain information

**Fig. 5:**
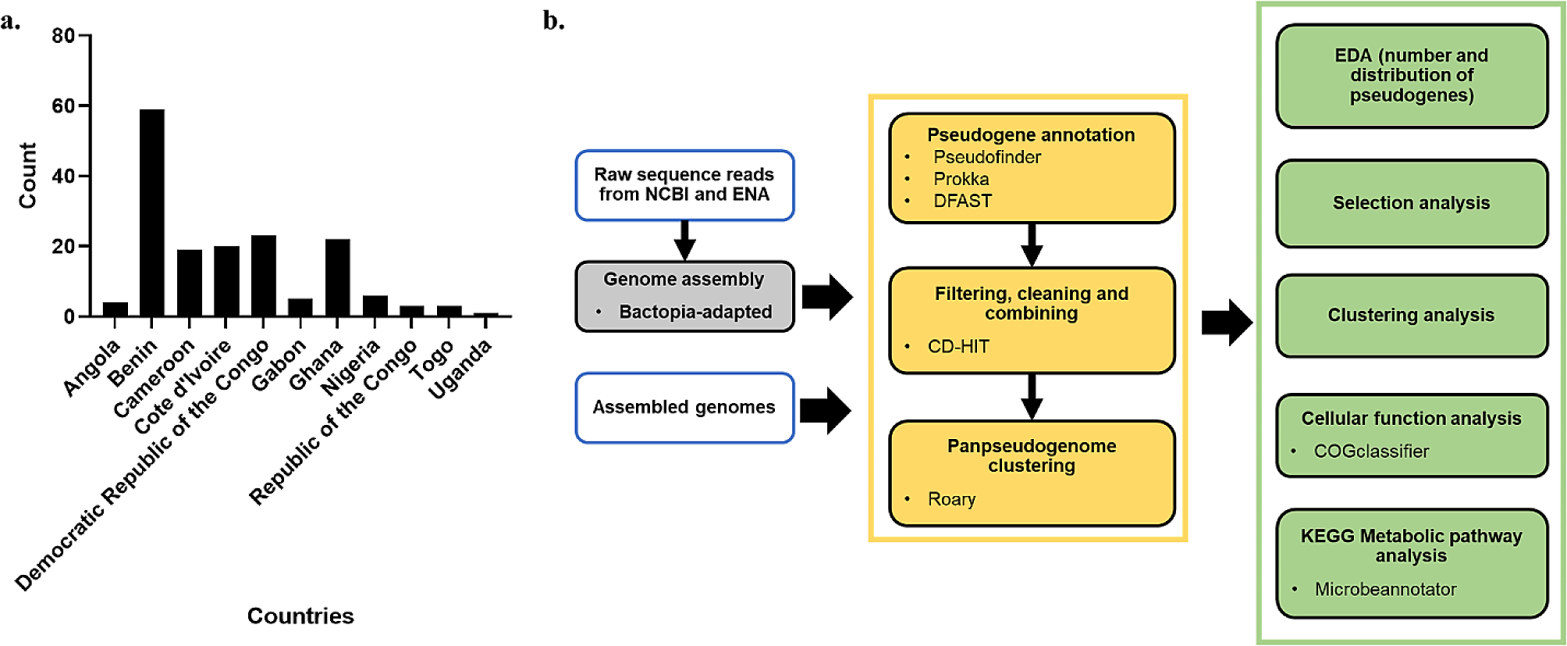
African MU strains and pseudogene analysis pipeline. (**a**) Distribution of *M. ulcerans* representing the African MU population [13]. (**b**) Pseudogene identification and analysis pipeline. *Blue boxes indicate inputs, yellow boxes indicate pseudogenome identification, and green boxes indicate pseudogenome comparative analysis*

In-house (PA38, Z007, and Z009) and African strains [13] were used in this sudy. In-house strains were collected from 2018 to 2019 from patients reporting to the clinic with lesions that were characteristic of Buruli ulcer lesions and confirmed with IS2404 PCR. PA38 is from Pakro in Ghana, and Z007 and Z039 are Ivorian strains from Zoukougbeu. The African strains consist of 165 clinical MU isolates from 11 countries (Fig. 5a) collected over 48 years (1964–2012). The method of sampling can be inferred that the isolates were obtained from patients reporting to the clinic. Agy99 reference strain is a clinical isolate from Ghana and served as a reference to all the MU strains. Non-African clinical strains from French Guiana and the USA were included in this study for comparison. *M. marinum* M reference strain (USA) was also included for comparison to MU strains.

### Pipeline development

#### Genome assembly

Whole genome data were obtained from the National Center for Biotechnology Information (NCBI) except for in-house genomes. The assembled genomes of PA38, Z007, and Z009 (Gyamfi et al., unpublished data) were sequenced with the Nanopore platform. The Illumina platform was used for the unassembled genomes of the African strains (PRJNA313185) and the Australian isolate (Biosample SAMN00992193 from PRJNA163311). The assembled genome of the French Guiana strain (PRJEB30628) was sequenced using Nanopore and Illumina platforms, and that of the USA strain (PRJNA191796) was sequenced with the PacBio platform. The Applied Biosystems platform was used to sequence Agy99 (PRJNA16230) and *M. marinum* M (PRJNA16725). The non-African strains were used as outgroups for comparison to the African strains and in-house genomes.

To assemble the MU genomes, tools from Bactopia [40] were used with sequence reads (SRA) as input. Preprocessing was done with sketch to estimate genome (https://mash.readthedocs.io/en/latest/sketches.html), adapters were trimmed with bbduk, and errors were corrected with lighter. Read quality check was done with fastq-scan (https://github.com/rpetit3/fastq-scan) and fastqc (https://www.bioinformatics.babraham.ac.uk/projects/fastqc). The genomes were further assembled with shovill (https://github.com/tseemann/shovill) using skesa and misassembly correction was done with ragtag (https://github.com/malonge/RagTag). The corrected assembled contigs were scaffolded with Agy99 using ragtag. The quality of assembly was assessed with checkm (Parks et al. 2015) and U50 [41] to determine genome contamination and completeness.

#### Pseudogene prediction

Prokka [17], DFAST [11] and Pseudofinder [18] were used to identify pseudogenes from pipeline-assembled and already-assembled whole genomes. Pseudogenes were combined and cleaned with CD-HIT to remove duplicate pseudogenes [12] (Fig. 5b). Roary [8] was further used to cluster pseudogenes and eliminate any additional duplicated pseudogenes generated from the use of different pseudogene prediction tools to finally generate the pseudogenome of each MU strain. The pseudogenome of the isolates was written into various file formats (protein and nucleic acid fasta and GeneBank flat file version 3 files) for downstream analysis.

#### Pseudogenomic analysis

Written and Roary files, from the pseudogene annotation and cleaning step, were used for exploratory data analysis (EDA) (Fig. 5b). The distribution of pseudogenes across isolates as well as by country was plotted with seaborn (https://github.com/mwaskom/seaborn). The distribution of pseudogenes across isolates employed the use of a binning approach that combined Freedman-Diaconis’s Rule and Shimazaki & Shinomoto’s method [42] to optimize the selection of bin size. Pearson’s correlation was calculated to determine the correlation between pseudogene accumulation across African MU strains with time.

To further understand pseudogenome dynamics across strains, selection pressure (ratio of synonymous and non-synonymous SNPs (dN/dS)) on pseudogenes was determined using Pseudofinder. For comparison, dN/dS was estimated for the whole genome and the functional genome (whole genome without pseudogenes) using the Basic Local Alignment Search Tool (BLAST) [43]. Isolates were further clustered based on pseudogenomes using hierarchical clustering and Uniform Manifold Approximation and Projection (UMAP) [44] for low-dimensionality representation. In using UMAP, different parameters (metric, number of neighbours, and the number of components) were tested and the optimum was used while using the hierarchical cluster to inform optimum representation. All assessed metrics of the pseudogenomes of the isolates were used to assess the evolution of the MU strains.

The impact of pseudogenization on the cellular processes and metabolism of the functional genome were determined using COGclassifier (https://github.com/moshi4/COGclassifier) and Microbeannotator [45] respectively. Bar graphs and heatmaps were used for visualization, comparing the whole genome and the functional genome. Comparisons made were used in generating a pseudogene-based niche model of MU. Finally, conserved pathways across the isolates were assessed for viability as possible drug targets.

#### Genomic analysis

The similarity of the scaffolded genomes was assessed using the average nucleotide identity calculated by dRep [46]. This is achieved by generating pairwise comparisons of the similarities in genomes using Mash [47]. The similarity cutoff for MU was set at 98.5% [5] with *M. marinum* M as an outgroup. dRep was executed with default parameters and comparisons were further graphed with hierarchical plots.

To assess the phylogenetic relationships among the MU strains, parsnp [48] was used (default parameters) to identify single nucleotide polymorphisms (SNPs) occurring in the core genome. The SNPs called were used to construct a maximum likelihood estimate tree with fastTree2 [37] and the exported tree in newick format was visualized and edited with ITOL [49]. Trees branches with bootstrap greater than 50% were maintained with branches depicting the level of substitutions in the core genome.

### Electronic supplementary material

Below is the link to the electronic supplementary material.

Supplementary Material 1

## Data Availability

The datasets analysed during the current study are available in the NCBI repository. The accession number(s) can be found at https://www.ncbi.nlm.nih.gov/sra/PRJNA812065; PRJNA163311; PRJEB30628; PRJNA191796; PRJNA16230; and PRJNA16725. Pseudopipe is implemented in Shell and is freely available at https://github.com/mosilab/pseudopipe under the BSD-3-Clause License which analyses KEGG data. The authors have obtained permission to include this information in the manuscript from www.kegg.jp/kegg/kegg1.html.

## Abbreviations

BU: Buruli ulcer
MU: *Mycobacterium ulcerans*
DDBJ: DNA Data Bank of Japan
ANI: Average Nucleotide Identity
UMAP: Uniform Manifold Approximation and Projection
CDS: Protein coding sequence
COG: Clusters of Orthologous Genes
KEGG: Kyoto Encyclopedia of Genes and Genomes
UV: Ultraviolet
VNTR: Variable Nucleotide Tanden Repeat
EDA: Exploratory data analysis
dN/dS: Non-synonymous/ synonymous
BLAST: Basic Local Alignment Search Tool
UMAP: Uniform Manifold Approximation and Projection
SNP: Single nucleotide polymorphisms
