# Supplementary material for: Pseudogenomic insights into the evolution of Mycobacterium ulcerans
Source: BMC Genomics. 2024 Jan 22;25:87. doi: 10.1186/s12864-024-10001-1 (PMC10802024; doi:10.1186/s12864-024-10001-1)
Supplement: Supplementary file 1 — Supplementary Material 1 [file 12864_2024_10001_MOESM1_ESM.docx]

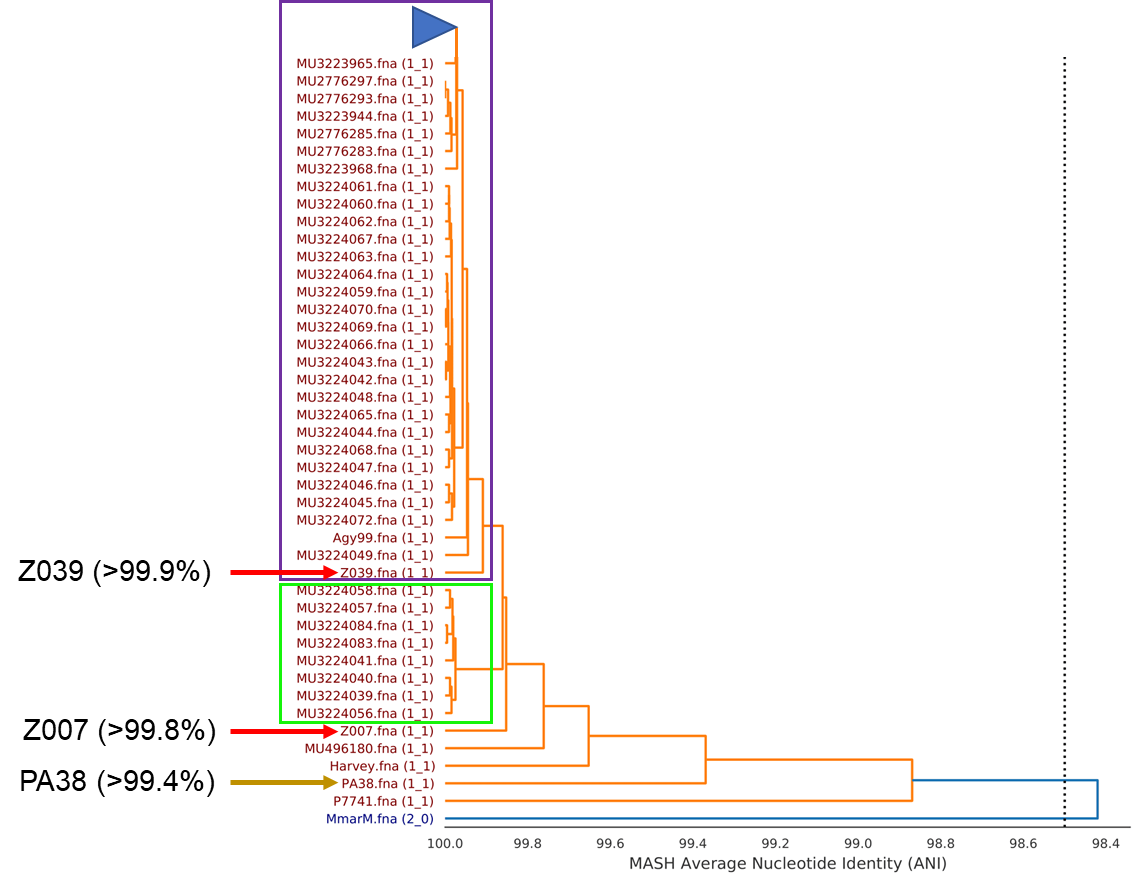


**Supplementary Figure S1: Average Nucleotide Identity (ANI)-based clustering of MU and MM strains using dRep.** Different species are coloured differently (MU in orange and MmarM in blue) using a percentage species identity cutoff of 98.5% (black broken line). Purple and green boxes represent prominent clades identified and the in-house strains are labelled with red and brown arrows indicating Ivorian and Ghanaian isolates respectively.

**
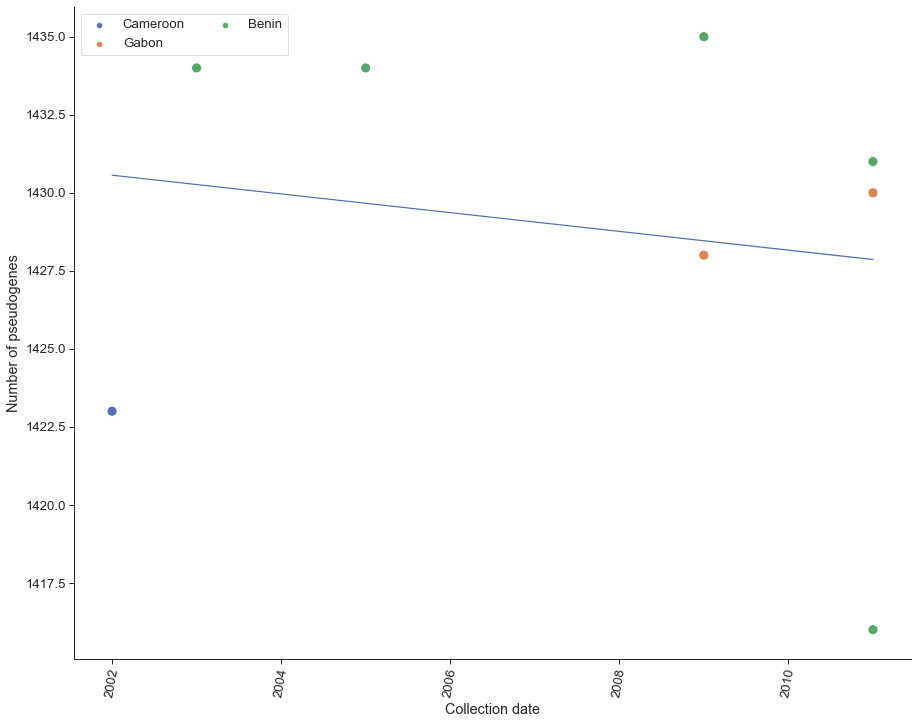
**

**Supplementary Figure S2: Correlation between sample collection date and the number of pseudogenes in lineage two African strains.** The blue line is a regression line with a negative Pearson’s rank correlation, r (8) = 0.172 (p-value = 0.683), and a gradient of -0.30 pseudogenes per year. Points are coloured by the source (country) of the strains.

**
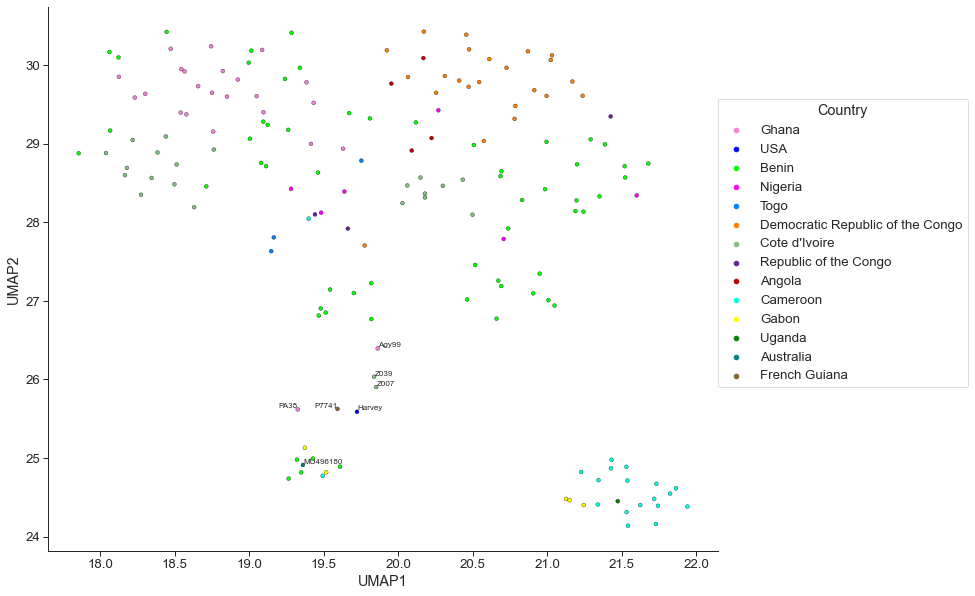
**

**Supplementary Figure S3: UMAP clustering of MU pseudogenomes with a n_neighboours of 200.** Isolates are coloured by country. Labelled isolates are in-house and non-African strains.


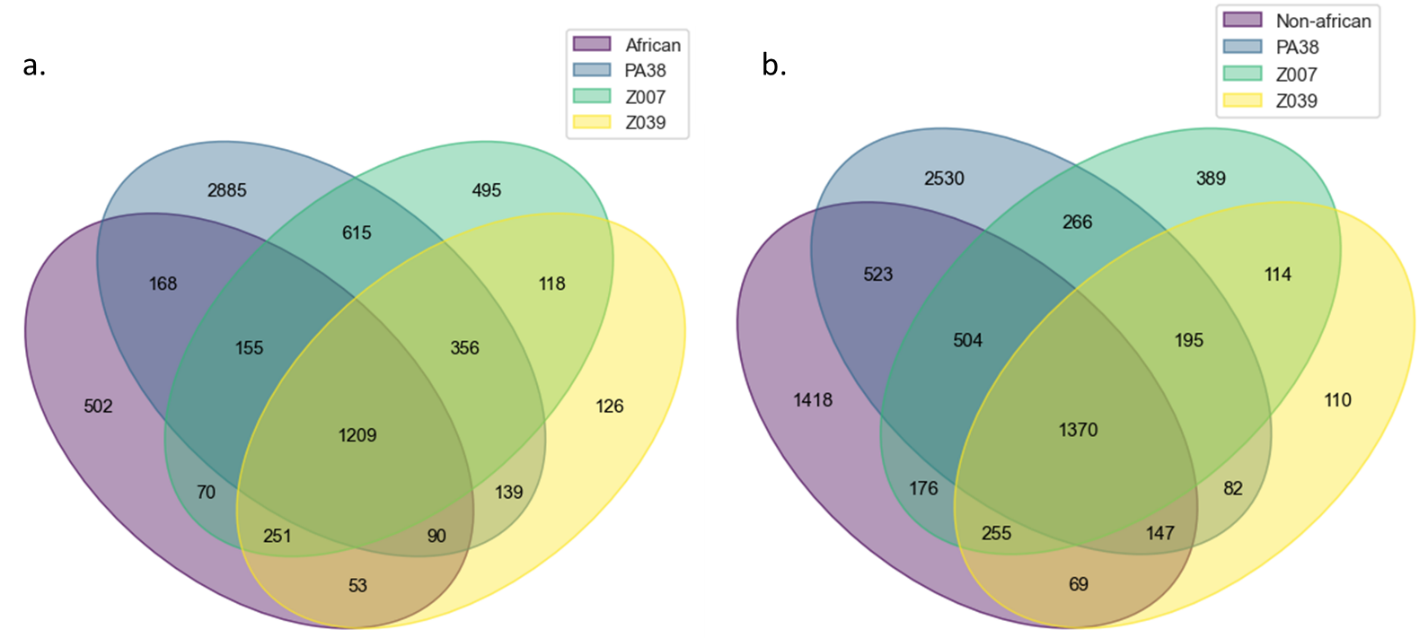


**Supplementary Figure S4: Venn diagram comparing pseudogenes in the African, non-African, and in-house MU isolates.** Comparison between a) the African group of strains and the in-house strains, and b) between non-African and in-house isolates.


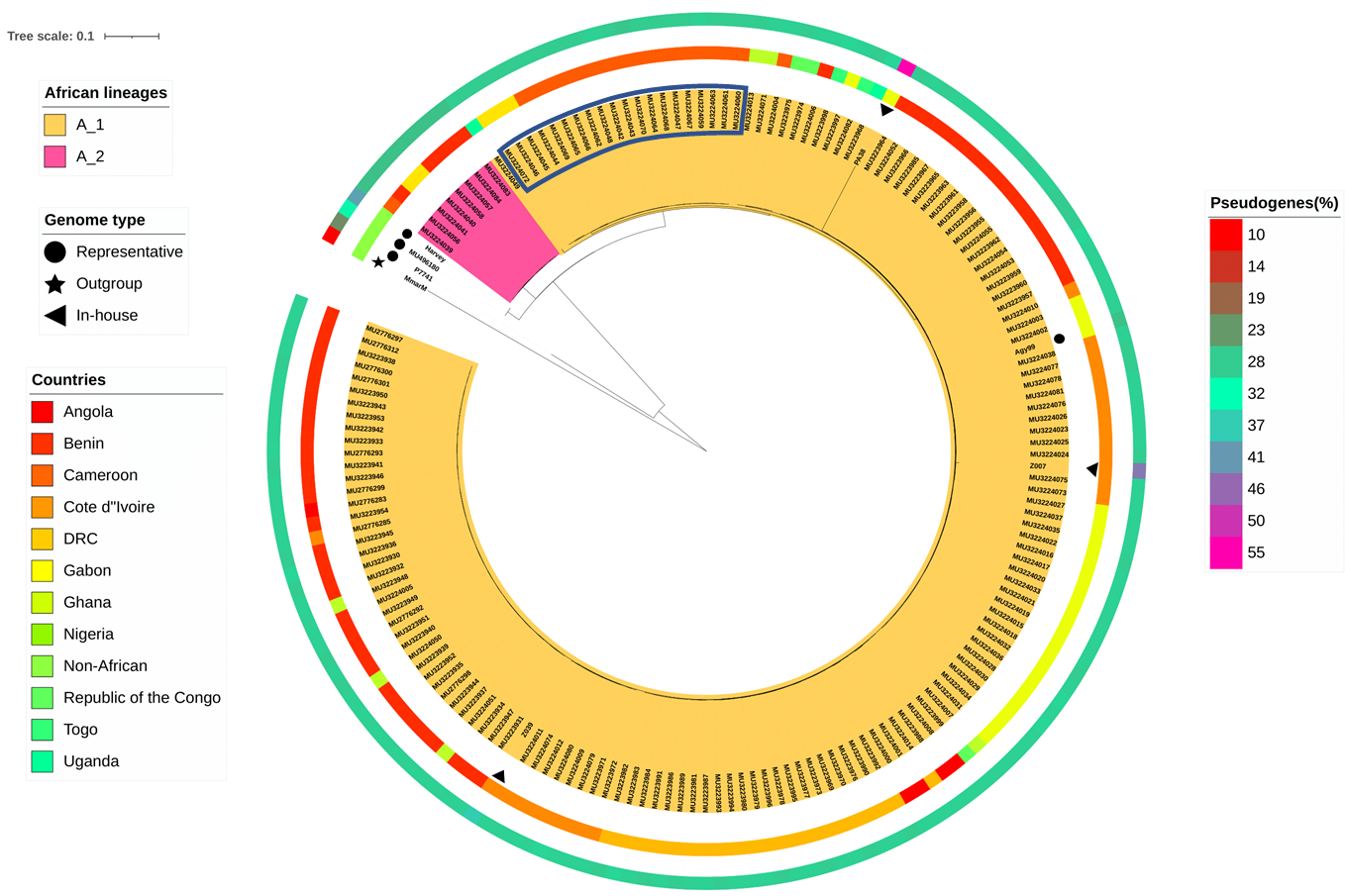


**Supplementary Figure S5: Phylogwntic analysis of MU genomes.** Maximum likelihood estimates phylogenetic tree of MU strains. MU strains include in-house (triangle) and non-African strains (circular), and MmarM (star) as an outgroup for comparison. Orange and purple coloured clades correspond to both lineage 1 and 2 respectively. The blue box marks the smaller subpopulation of isolates from Gabon and Cameroon. The outer circle represents the source(country) of the isolate.


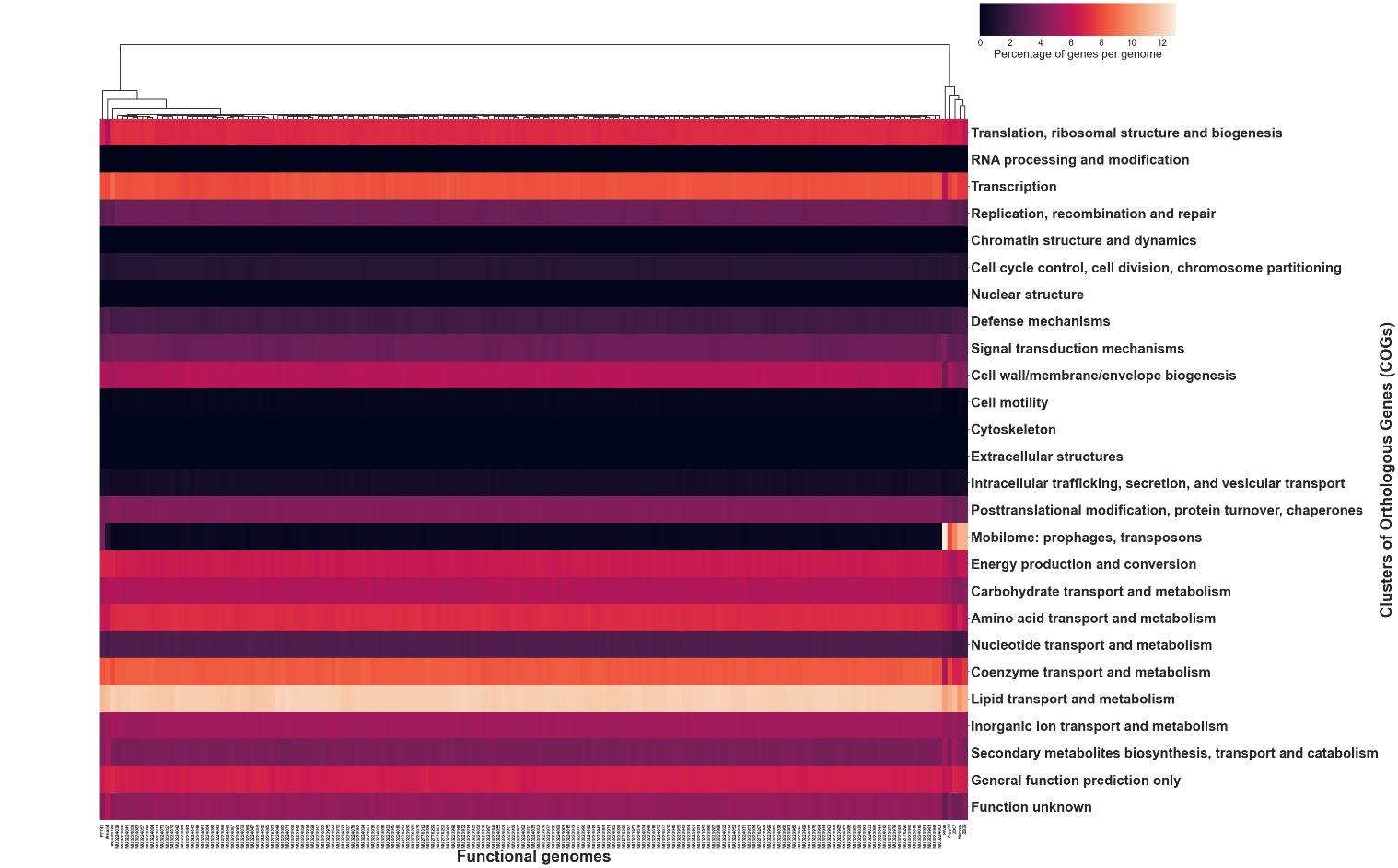


**Supplementary Figure S6: Cellular processes of *M. ulcerans* functional genomes using the COG database.** The hierarchical cluster represents similarities in COG patterns across MU strains. Colours represent the percentage of pseudogenes per pseudogenome.


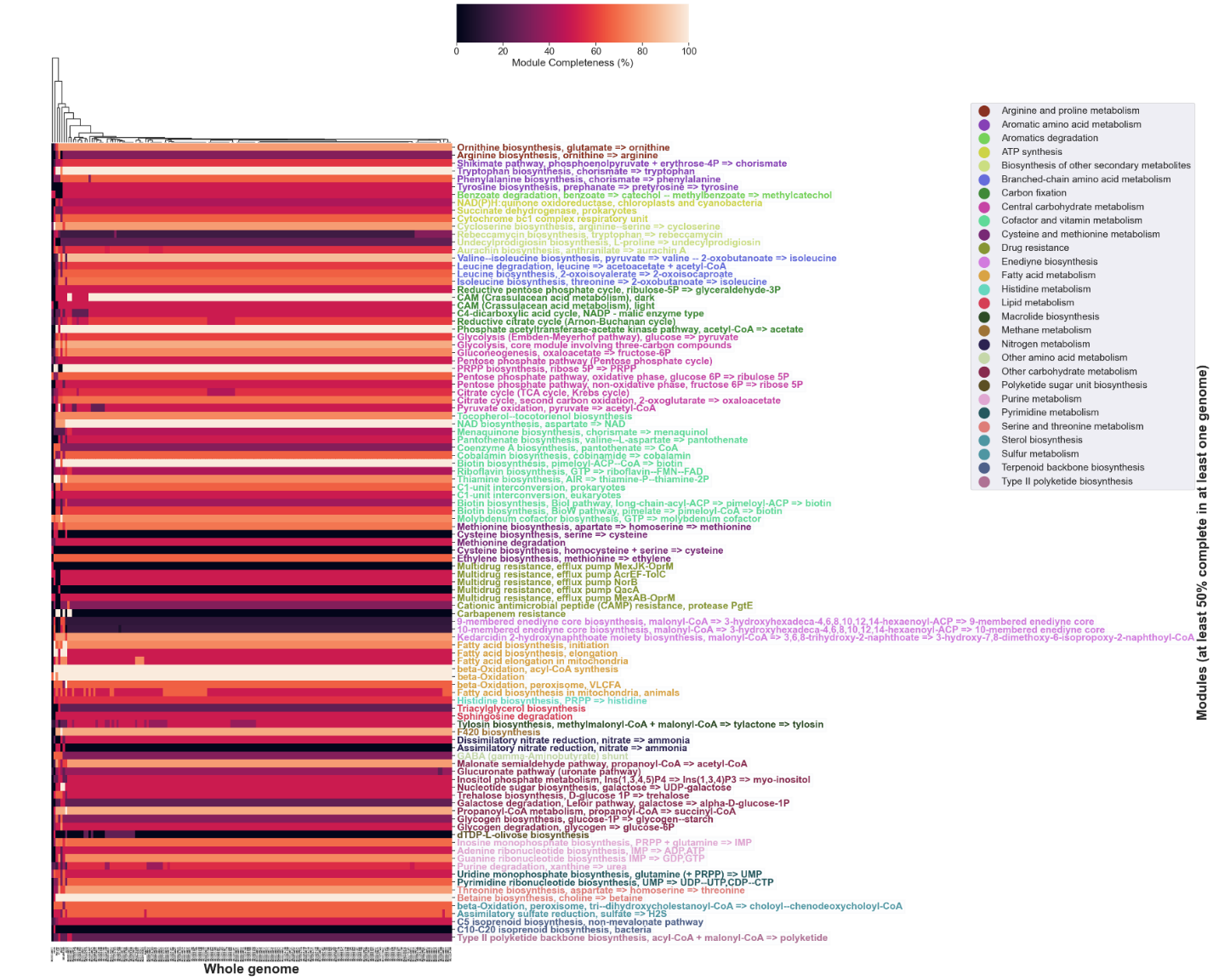


**Supplementary Figure S7: KEGG metabolic pathway analysis of *M. ulcerans* whole genomes.** Pathway modules are coloured based on the broader metabolism groups and each pathway module has an *M. ulcerans* strain that shows at least ≥50% module completeness. The hierarchical cluster represents similarities in metabolic patterns across MU strains.


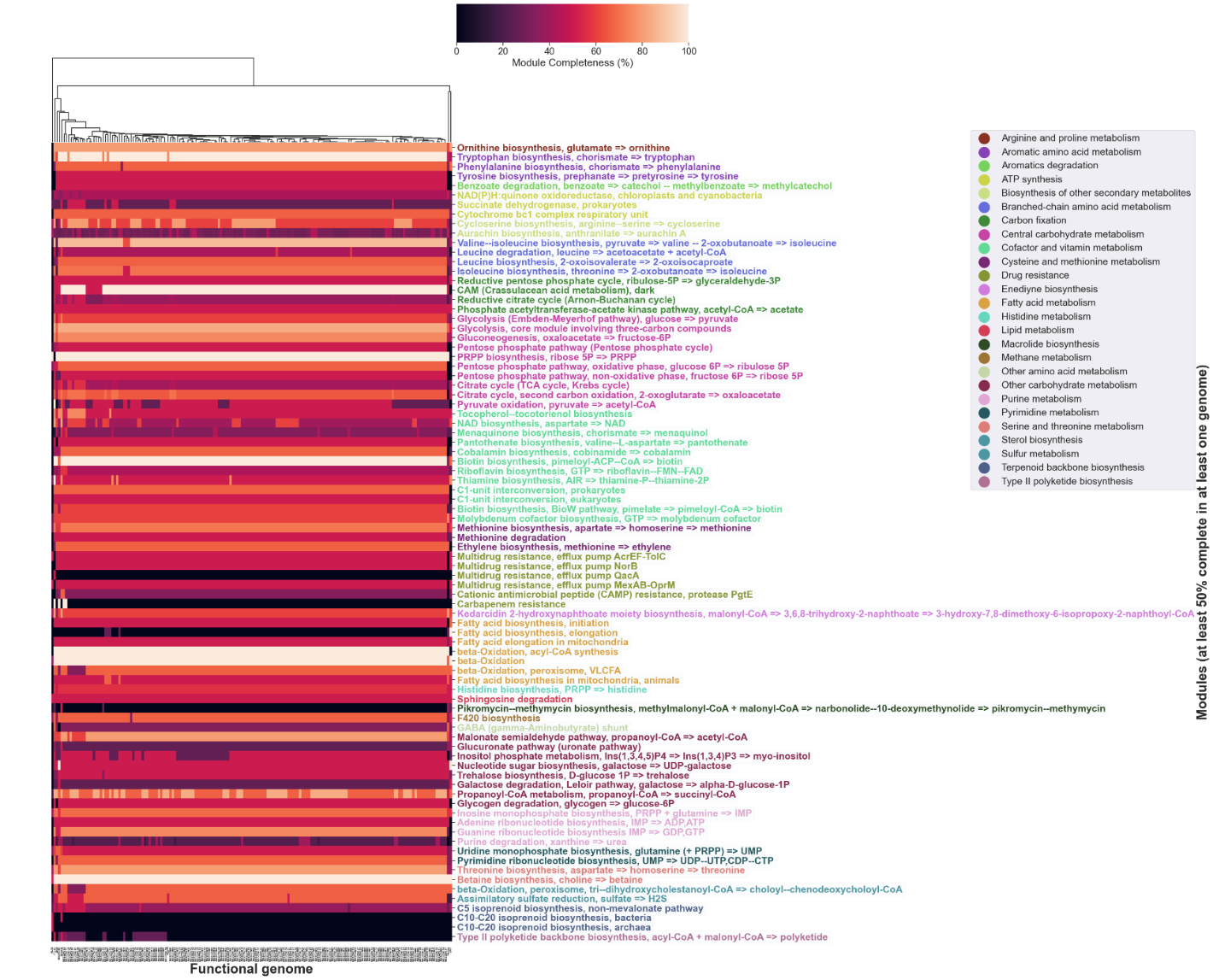


**Supplementary Figure S8: KEGG metabolic pathway analysis of *M. ulcerans* functional genomes.** Pathway modules are coloured based on the broader metabolism groups and each pathway module has an *M. ulcerans* strain that shows at least ≥50% module completeness. The hierarchical cluster represents similarities in metabolic patterns across MU strains.

**Supplementary Table S1: KEGG metabolic pathway variations in functional genome compared to the whole genome.**

| **Metabolism absent** | **Pathway module absent** | **Pathway module decrease/variation** |
| --- | --- | --- |
| *Nitrogen metabolism  *Polyketide sugar unit biosynthesis | *Arginine biosynthesis, ornithine => arginine  *Shikimate pathway, phosphoenolpyruvate + erythrose-4P => chorismite  *Rebeccamycin biosynthesis, tryptophan => rebeccamycin  *Undecylprodigiosin biosynthesis, L-proline => undecylprodigiosin  *CAM (Crassulacean acid metabolism), light  *C4-dicarboxylic acid cycle, NADP - malic enzyme type  *Coenzyme A biosynthesis, pantothenate => CoA  *Biotin biosynthesis, BioI pathway, long-chain-acyl-ACP => pimeloyl-ACP => biotin  *Cysteine biosynthesis, serine => cysteine  *Cysteine biosynthesis, homocysteine + serine => cysteine  *Multidrug resistance, efflux pump MexJK-OprM  *9-membered enediyne core biosynthesis, malonyl-CoA => 3-hydroxyhexadeca-4,6,8,10,12,14-hexaenoyl-ACP => 9-membered enediyne core  *10-membered enediyne core biosynthesis, malonyl-CoA => 3-hydroxyhexadeca-4,6,8,10,12,14-hexaenoyl-ACP => 10-membered enediyne core  *Triacylglycerol biosynthesis  *Tylosin biosynthesis, methylmalonyl-CoA + malonyl-CoA => tylactone => tylosin  *Dissimilatory nitrate reduction, nitrate => ammonia  *Assimilatory nitrate reduction, nitrate => ammonia  *Glycogen biosynthesis, glucose-1P => glycogen--starch | *Succinate dehydrogenase, prokaryotes  *Cycloserine biosynthesis, arginine--serine => cycloserine  *Aurachin biosynthesis, anthranilate => aurachin A  * Leucine degradation, leucine => acetoacetate + acetyl-CoA  *Reductive citrate cycle (Arnon-Buchanan cycle)  *Citrate cycle (TCA cycle, Kreb cycle)    *Pyruvate oxidation, pyruvate => acetyl-CoA  *Tocopherol--tocotorienol biosynthesis  *NAD biosynthesis, aspartate => NAD  * Thiamine biosynthesis, AIR => thiamine-P--thiamine-2P  *Kedarcidin 2-hydroxynaphthoate moiety biosynthesis, malonyl-CoA => 3,6,8-trihydroxy-2-naphthoate => 3-hydroxy-7,8-dimethoxy-6-isopropoxy-2-naphthoyl-CoA  *Fatty acid biosynthesis, initiation  *Fatty acid biosynthesis, elongation  *Fatty acid elongation in mitochondria  *Fatty acid biosynthesis in mitochondria, animals  *F420 biosynthesis  *Malonate semialdehyde pathway, propanoyl-CoA => acetyl-CoA  *Glucuronate pathway (uronate pathway)  *Inositol phosphate metabolism, Ins(1,3,4,5)P4 => Ins(1,3,4)P3 => myo-inositol  *Propanoyl-CoA metabolism, propanoyl-CoA => succinyl-CoA  *Purine degradation, xanthine => urea  *beta-Oxidation, peroxisome, tri--dihydroxycholestanoyl-CoA => choloyl--chenodeoxycholoyl-CoA  *Assimilatory sulfate reduction, sulfate => H2S  *C5 isoprenoid biosynthesis, non-mevalonate pathway  *Type II polyketide backbone biosynthesis, acyl-CoA + malonyl-CoA => polyketide |
